# Clinical practice recommendations and expected outcomes with fluorescent light energy: a Delphi-like consensus

**DOI:** 10.1186/s12917-025-05047-6

**Published:** 2025-10-02

**Authors:** Luisa Cornegliani, Amelia White, Frédéric Sauvé, Laura Ordeix, Ursula Mayer, Courtney Campbell, Jacques Fontaine, Galia Sheinberg, Emmanuel Bensignor, Andrea Marchegiani, Becky Valentine, Oscar Fantini, Anthony Yu

**Affiliations:** 1 Clinica Veterinaria Città di Torino, Torino, 10135 Italy; 2 Department of Clinical Sciences, College of Veterinary Medicine, Auburn University, 1220 Wire Road, Auburn, AL USA; 3 Faculté de médecine vétérinaire, Université de Montréal, Saint- Hyacinthe, Québec, Canada; 4 Departament de Medicina i Cirurgia Animals, Fundació Hospital Clínic Veterinari, Universistat Autònoma de Barcelona, Barcelona, Spain; 5 AniCura Kleintierspezialisten Augsburg GmbH, Dermatology, Augsburg, Germany; 6 Stitches Veterinary Surgery Center, Long Beach, CA 90815 USA; 7 Faculté de Médecine Vétérinaire, Université de Liège, Liège, Belgium; 8 Dermatología Especializada, Centro Veterinario México, Mexico City, Mexico; 9 Department of Clinical Sciences, Dermatology Unit, Oniris Nantes Vet School, Nantes, F-44300 France; 10 School of Biosciences and Veterinary Medicine, University of Camerino, via Circonvallazione 93/95, Matelica, 62024 Italy; 11 Calgary Pet Dermatology Centre, PETDERM, Calgary, AB Canada; 12 Vetoquinol SA, Paris, France; 13 Veterinary Allergy Dermatology Ear Referral (V.A.D.E.R.) Clinic Morriston, Morriston, ON Canada

**Keywords:** Fluorescent light energy, Delphi-like method, Consensus, Veterinary dermatology, Skin diseases, Photobiomodulation

## Abstract

**Background:**

Fluorescent Light Energy (FLE) is a promising alternative to systemic therapies in veterinary dermatology and surgery for managing skin conditions and improving the quality of life of animals and their owners.

**Hypothesis/objectives:**

In the absence of specific recommendations for FLE use, an international DELPHI consensus research project was conducted to establish best practices.

**Methods:**

An international Steering Committee (SC) of a board-certified veterinary surgeon and veterinary dermatologists combined a literature review with clinical expertise to create recommendations. General practitioners and veterinarians of various specialties were selected to review and vote on the recommendations. Votes were collected electronically, independently, and anonymously.

**Results:**

The statements covering the following topics were analyzed in this paper: (i) Understanding photobiomodulation via FLE; (ii) Indications and Protocols for FLE; and (iii) FLE pet owner information. Consensus was reached on 33 out of 33 statements (100%) addressing the use of photobiomodulation via FLE; the practical modalities of FLE as monotherapy or adjunct therapy; healing biological benefits of photobiomodulation; reduction of antibiotic use in the management of bacterial skin infections; clinical indications where FLE can show the most favorable results along with protocols and duration of treatment; and communication with animal owners on safety measures and FLE’s benefits for their animal.

**Conclusions and clinical importance:**

This consensus provides practical guidelines on the utilization, application, and benefits of FLE when addressing veterinary dermatological conditions. It contributes to optimizing animal and owner welfare and bridges the gap between expert recommendations and the real-life experiences of general practice veterinarians.

**Supplementary Information:**

The online version contains supplementary material available at 10.1186/s12917-025-05047-6.

## Introduction

Skin diseases in companion animals are one of the most common complaints in veterinary general practice. These conditions compromise the well-being of animals and present a burden for both the owner and the veterinarian [1–3]. Greater severity of skin disease and increased treatment complexity tend to be associated with a higher animal owner burden that challenges the veterinarian–client-patient relationship (VCPR) [4–6]. Simplifying treatment regimens can help improve the quality of life of animals, promote the VCPR, and help improve owner adherence [7]. The pursuit of cutting-edge, non-invasive technologies to manage skin conditions has led to transformative breakthroughs, including the development of light-based therapies [8]. Photobiomodulation (PBM), formerly known as low-level light therapy (LLLT), has been explored for decades to promote tissue repair, reduce inflammation, and modulate pain. Notably, the National Aeronautics and Space Administration (NASA) investigated the use of light-emitting diodes (LEDs) in the 1990 s to enhance wound healing and tissue regeneration in microgravity environments [9]. Since then, PBM has found applications across regenerative medicine, dermatology, rehabilitation, and other clinical domains requiring modulation of healing and inflammation [10, 11].

Among the different PBM techniques developed for human and veterinary use, fluorescent light energy (FLE**)** has gained substantial interest. In FLE, a blue (440–460 nm) LED illuminates a topical photoconverter gel, producing polychromatic, low-energy visible light in the form of fluorescence. This light penetrates the skin to varying depths, interacting with multiple chromophores to stimulate cellular responses involved in healing, inflammation control, and, potentially, bacterial load reduction [8, 12–14].

Mechanistically, FLE appears to exert a multifactorial, host-mediated effect. At the mitochondrial level, it activates cytochrome c oxidase, enhancing oxidative phosphorylation and adenosine triphosphate (ATP) synthesis; ultrastructural analyses of treated lesions show substantial increases in mitochondrial number and volume with well-preserved cristae [15]. At the molecular level, FLE down-regulates pro-inflammatory cytokines (IL-1α, IL-1β, IL-6, IL-17, TNF-α), up-regulates anti-inflammatory mediators (IL-10, TGF-β), and promotes growth factor release (EGF, VEGF, FGF, PDGF, TGF-β), driving keratinocyte and fibroblast proliferation, as reflected by increased Ki-67 expression [8, 14–16]. The observed analgesic effect, although not fully elucidated, is likely mediated by attenuation of the inflammatory cascade responsible for peripheral sensitization, possibly complemented by modulation of nociceptor ion channels such as TRPV1, resulting in reduced activation thresholds [10, 11, 14, 15]. Blue light in the 400–470 nm range may also inhibit *Staphylococcus* spp. by photoexciting endogenous bacterial porphyrins, generating reactive oxygen species that damage cell membranes and DNA [10, 14–16]. Although not specifically demonstrated for FLE, veterinary clinical studies report efficacy in bacterial skin infections both as monotherapy and in conjunction with systemic antibiotics, with significantly shorter healing times than antibiotic therapy alone [8]. This property supports a reduction in antimicrobial use, highly relevant in the context of global antimicrobial resistance [17–19].

Beyond lesion resolution, the adherence, the veterinarian–client–patient relationship (VCPR), and the quality of life are also important. In a 2023 prospective study by Mosca et al. [20], 35 dogs diagnosed with deep pyoderma, interdigital furunculosis, pyotraumatic dermatitis, perianal fistulas, or surgical/traumatic wounds were treated with FLE. QoL was evaluated using a validated 15-item owner questionnaire assessing overall disease severity as well as seven domains each of canine and owner well-being. Most dogs achieved complete remission. Owners also reported better quality of life (QoL), with 74% of dogs showing ≥ 50% improvement in total QoL scores.

To date, all published veterinary studies on FLE, demonstrating the effect in various conditions including interdigital furunculosis, superficial and deep pyoderma, perianal fistulas, pyotraumatic dermatitis, and acute or chronic wounds [8] have used the Phovia^®^ system (Phovia^®^, Vetoquinol, France). This system comprises a blue LED light source and a topical photoconverter gel containing light-absorbing chromophores, which, when illuminated, emit fluorescent light capable of eliciting photobiomodulatory effects [21].

The benefits of FLE in veterinary dermatology have been described widely in the literature, but to the best of our knowledge, a consensus on the usage, application, and benefits of FLE has not been established.

The objectives of this study were to assess current practices for the use of FLE delivered via the Phovia^®^ system, which to date is the only commercially available veterinary device using this technology in veterinary medicine and to identify areas of consensus amongst an international group of veterinarians on a wide range of recommendations generated by an international Steering Committee (SC) composed of a board-certified veterinary surgeon and a group of veterinary dermatologists. To this end, we employed a modified Delphi approach to forge a consensus and bridge the gap between expert guidelines and recommendations and the real-world experiences of general practitioners.

## Methods and materials

**Fig. 1:**
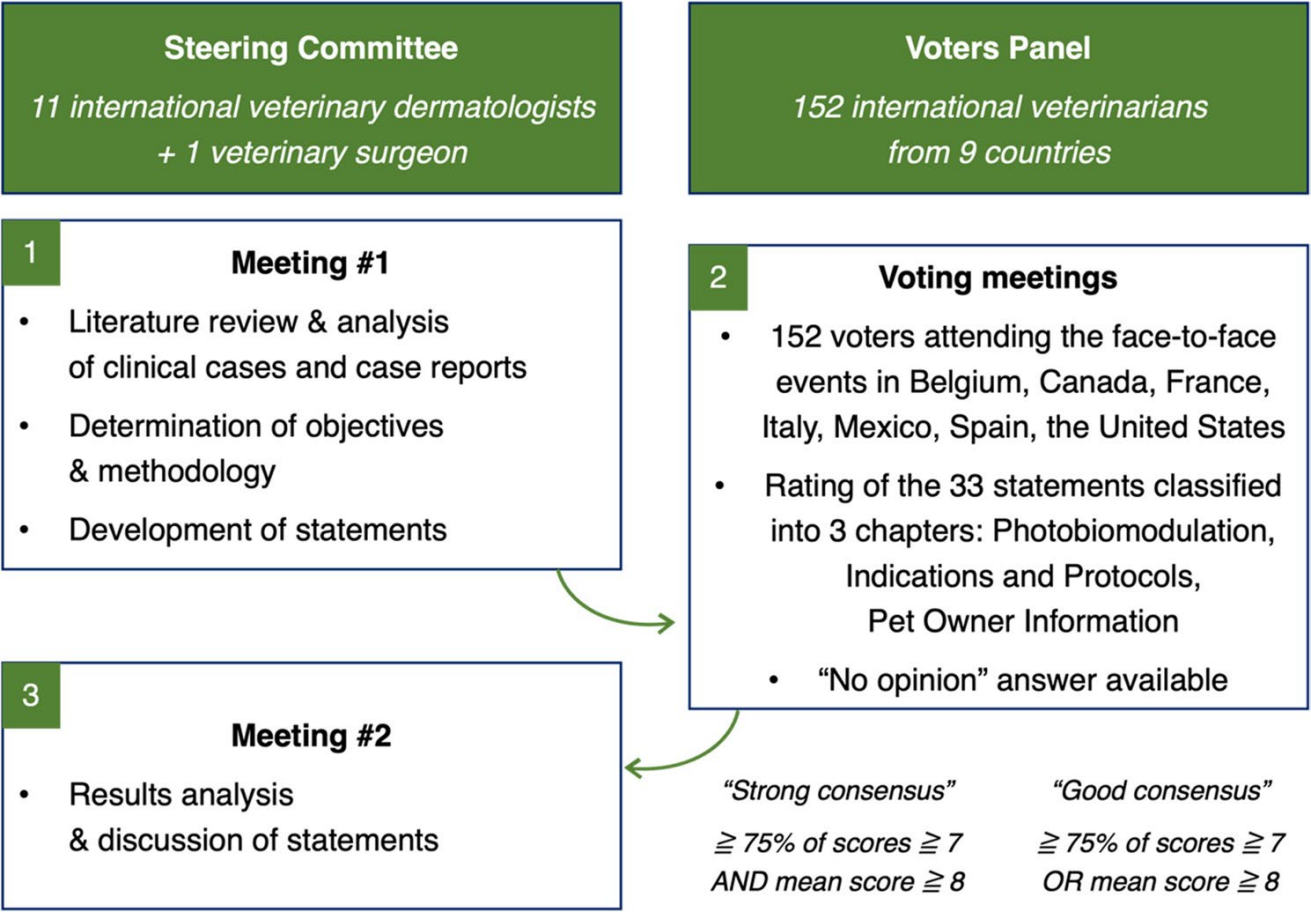
The modified Delphi method used in this study

The Delphi method is an iterative consensus approach based on information collected from a panel of voters who have various degrees of knowledge in the subject under consideration and who consistently treat patients with conditions thereof [22–26]. This method has been used extensively in many human therapeutic areas and several times in veterinary dermatology [27–29]. In this structured approach, voters give their opinions individually and anonymously to express their degree of agreement on several statements to achieve consensus on a specific and well-defined subject. In accordance with international methodologies [22, 23], the present study was structured as a modified international Delphi consensus conducted with general practitioners and veterinarians of different specialties from nine countries between October 2023 and January 2024. The voters’ opinions were gathered during the organization of seven face-to-face meetings in Belgium, Canada, France, Italy, Mexico, Spain, and the United States using a questionnaire developed by a SC composed of international experts (Fig. 1). Voters specified their level of agreement with each statement using a nine-point Likert scale ranging from one “Strongly disagree” to nine “Strongly agree” [30]. The percentage of scores and the median were calculated for each statement. A *strong consensus* was reached for a statement when at least 75% of the scores were ≥ 7 and the median score was ≥ 8. When only one of these two parameters was satisfied, the statement was considered to have obtained a *good consensus* [22, 30].

### Steering committee and statements

The SC comprised 12 international experts: 11 veterinary dermatologists and one veterinary surgeon. Based on a thorough analysis of the literature, case reports, and their clinical experience, 46 statements on the use of FLE and its benefits in veterinary dermatology were developed over the course of two SC meetings during the summer of 2023. These statements were divided into five chapters: Photobiomodulation; Indications and Protocols; Pet Owner information; Practice Organization; and Business Considerations.

### Voting groups

To be considered as a voter, each participant at one of the seven American or European events had to have used FLE (Phovia^®^, Vetoquinol, France) on at least one patient. The outcome of this intervention, whether favorable or not, was not considered as an inclusion factor to avoid bias towards FLE users who might have only experienced positive outcomes in their clinics. All voters also had to physically attend one of the seven consensus meetings for their entire duration.

Each consensus meeting consisted of five plenary sessions featuring the latest published data in the field, clinical cases drawn from either literature or the speakers’ recent patient encounters, and demonstrations of FLE (Phovia^®^, Vetoquinol, France) implementation in the speakers’ clinics with one session dedicated to each of the aforementioned chapters. Each voting session was thus preceded by a presentation by one or several experts from the SC. Voting was conducted electronically on the voters’ cell phones or other hand-held devices linked to polling software (mec.radiant-ia.tech™, France) developed specifically for the events. While voters were allowed to ask questions to clarify language-related issues on certain statements during the events, questions and answers were kept to a minimum to avoid voters biasing their peers’ answers at the time of voting. There was a single round of voting for each consensus meeting and no possibility for voters to develop or explain their opinions for each statement before a second round of voting. A “no opinion” response option was included for participants who could not answer a statement. The “no opinion” votes were excluded from the final analysis for each statement.

Any personal data transmitted for the study was separated from the results and anonymized. The anonymity of the voting groups was always guaranteed during the voting. The SC members did not take part in the voting.

### Statistical analysis

In accordance with Delphi methodology, no formal power-based sample size calculation was performed, as the primary objective was to assess consensus rather than to test inferential hypotheses. The expert validation panel consisted of 12 members, selected to provide a balanced representation of expertise within the range recommended in the literature. The subsequent voting phase included all eligible participants who agreed to contribute across multiple countries.

Individual votes on a 9-point Likert scale (1 = strongly disagree, 9 = strongly agree) were collected electronically and analyzed by calculating the distribution of responses across three validity bands (1–3, 4–6, 7–9), the proportion of high-agreement scores (≥ 7), and the median score as a measure of central tendency. All statistical analyses were performed using SAS v9.1 (SAS Institute Inc.,)

## Results

### Participants

One hundred and fifty-two voters participated in the consensus meetings. Slightly more than half of the attendees (55%, *n* = 84) originated from the Americas, with the remaining 45% (*n* = 68) originating from Europe (Table 1). Most voters (73%, *n* = 111) were general practitioner veterinarians. Nearly all participants reported treating dogs (99%, *n* = 150) and most participants reported treating cats 84% (*n* = 127) in their practices (Table 1). Within this sample group, the median experience with the Phovia^®^ system was five animals treated per month (interquartile range or IQR [2–8]), (Table 1). Further characteristics of the participants are summarized in Table 1.

**Table 1:** Demographics and practice characteristics of voters (*N*=152)

Characteristic	All veterinarians
Total sample, *N* (%)	152 (100)
Location of practice, *N* (%)	
Mexico	36 (23)
Canada	27 (18)
The United States	21 (14)
Italy	19 (12)
Belgium	18 (12)
Spain	15 (10)
France	12 (8)
The Netherlands	3 (2)
Portugal	1 (1)
Expertise, *N* (%)	
General practitioner	111 (73)
veterinarians	26 (17)
Veterinary dermatologists	7 (5)
Veterinary surgeons	8 (5)
Others	
Number of animals treated with FLE (Phovia^®^ system, Vetoquinol) per month, median [IQR]	5 [2–8]
Types of animals treated, *N* (%)	
Dogs	150 (99)
Cats	127 (84)
Exotics	32 (21)
Horses	8 (5)
Others	14 (9)

*FLE* Fluorescent Light Energy, *IQR* interquartile range

### Statements

A *strong consensus* (≥ 75% of votes ≥ 7 and median ≥ 8) was reached for all 33 statements. The distribution of votes, medians, and results are provided in Tables 2, 3 and 4. The range of consensus was 81 to 99.3%. Twenty-six statements had a consensus above 95%, four had a consensus between 90% and 95%, and three statements had a consensus between 80% and 90%.

**Table 2:** Statements and voting results of the *Photobiomodulation* chapter

Statements	Validity 1-2-3 (*N*)	Validity 4-5-6 (N)	Validity 7-8-9 (*N*)	No opinion	Median	Results
*Photobiomodulation*						
1. Photobiomodulation *via* fluorescent light energy in veterinary skin disorders is a recommended supportive therapeutic approach for its regenerative, anti-inflammatory, and likely antimicrobial effects.	0% (0)	5.6% (7)	94.4% (119)	0	9	Strong consensus
2. Use of a chromophore gel with blue LED helps to promote the production of fluorescent light energy with a broader spectrum of visible light (440 to 700 nm) that penetrates to greater depths in the skin (up to approximately 6 mm), providing a broader range of effects.	3.2% (4)	1.6% (2)	95.2% (119)	3	8	Strong consensus
3. In the skin, light absorption generated by fluorescent light energy depends on the interaction with the different chromophores (e.g. cytochrome C oxidase, flavins, opsins) which absorb specific wavelengths to exert their biologic effects.	0% (0)	0.8% (1)	99.2% (120)	6	9	Strong consensus
4. Veterinary practitioners should be encouraged to use photobiomodulation via fluorescent light energy to promote clinical outcomes and reduce the length of systemic antibiotic use in patients with bacterial pyoderma, including patients with MRSP.	0% (0)	3.6% (4)	96.4% (106)	2	9	Strong consensus
5. Photobiomodulation via fluorescent light energy can be used as a monotherapy or as an adjunct to support other therapeutic regimens to manage non-neoplastic dermatologic conditions.	0% (0)	1.6% (2)	98.4% (127)	1	9	Strong consensus
6. Photobiomodulation via fluorescent light energy is recommended to be used early in the disease process to maximize efficacy.	0% (0)	3.4% (4)	96.6% (113)	9	9	Strong consensus
7. For the most favorable results using photobiomodulation via fluorescent light energy: • Illuminate the treated area with a licensed LED lamp delivering a peak wavelength between 440-460 nm, with a power density of 55-129 mW/cm^2^ for 2 minutes at a maximum distance (~ 5 cm). • Illuminate twice weekly or once weekly with 2 consecutive applications with cleaning and reapplication of new chromophore gel in between.	0% (0)	0.8% (1)	99.2% (122)	6	9	Strong consensus
8. To maximize efficacy and increase the likelihood of photonic penetration during photobiomodulation via fluorescent light energy, it is recommended to clip hair from treatment areas.	0% (0)	4.8% (6)	95.2% (119)	6	9	Strong consensus
9. To achieve optimal effects, photobiomodulation via fluorescent light energy should be used with a photoconverter gel uniformly applied to an approximately 2 mm layer of the treatment areas.	0% (0)	0.7% (1)	99.3% (134)	2	9	Strong consensus
10. Once fluorescent light energy treatment is completed, the photoconverter gel must be cleansed from the treated site(s).	4.6% (6)	9.2% (12)	86.2% (112)	3	9	Strong consensus
11. When initiating fluorescent light energy treatment, a dermatologic workup is recommended to identify and treat all underlying causes and associated factors.	0.8% (1)	4.6% (6)	94.6% (124)	3	9	Strong consensus
12. An incomplete response to fluorescent light energy after 2 weeks of treatment should prompt a reevaluationof the underlying etiology and the therapeutic regimen.	6.3% (8)	12.7% (16)	81% (102)	1	9	Strong consensus
13. Photobiomodulation via fluorescent light energy treatment for a surface area exceeding the lamp diameter will require multiple treatments, increasing the length of time per session, or the use of multiple lamps being used simultaneously to keep session lengths to a minimum.	1.5% (2)	6.1% (8)	92.4% (121)	6	9	Strong consensus
14. Fluorescent light energy is painless; sedation may be required in aggressive or anxious patients.	4.3% (5)	8.7% (10)	87% (100)	3	9	Strong consensus

The variability in the total number of voters (equal or below 152) for each statement accounts for the use of the “no opinion” option by voters and for veterinarians who may not have submitted their answer in time before the voting process was closed

*FLE* Fluorescent Light Energy, *MRSP* methicillin-resistant *Staphylococcus pseudintermedius,* *LED* light emitting diode

**Table 3:** Statements and voting results of the *Indications and Protocols* chapter

Statements	Validity 1-2-3 (*N*)	Validity 4-5-6 (*N*)	Validity 7-8-9 (*N*)	No opinion	Median	Results
Indications and Protocols						
15. Photobiomodulation via fluorescent light energy is recommended for skin infections, wounds, and chronic and recurrent inflammatory skin conditions as it promotes tissue healing, reduces inflammation, and supports collagen production.	0% (0)	0.8% (1)	99.2% (132)	0	9	Strong consensus
16. Based on scientific studies* and case reports**, Phovia^®^ as a monotherapy or adjunct treatment is recommended for the following indications^†^: interdigital pyoderma*; surgical wounds*; deep pyoderma*; superficial pyoderma*; inflammatory perianal furunculosis (“fistula”)*; acute traumatic and chronic wounds*; acute pyotraumatic dermatitis* ^*†*^*Listed by level of evidence*	0% (0)	3% (4)	97% (131)	0	9	Strong consensus
17. Based on the experience of a group of veterinary dermatologists, Phovia^®^ can also be used as a monotherapy or adjunct therapy in the following indications: callus dermatitis; scrotal dermatitis; fold dermatitis (intertrigo), acral lick dermatitis.	0% (0)	3% (4)	97% (133)	1	9	Strong consensus
18. Based on single or few cases, Phovia^®^ treatment may be indicated in the following conditions but requires further investigation: equine pastern dermatitis (pyoderma, leucocytoclastic vasculitis); actinic dermatitis; feline idiopathic ulcerative dermatitis; cutaneous vasculitis (ear tip vasculitis); marsupialized abscesses; follicular dysplasia (alopecia X, pattern alopecia, post-clipping alopecia, seasonal flank); calcinosis cutis; pre- and post-surgical wounds (skin grafts, skin flaps, traumatic injuries).	0% (0)	6% (8)	94% (127)	5	9	Strong consensus
19. It is recommended that Phovia^®^ 2-minute sessions be applied twice weekly or once weekly with 2 consecutive applications.	0% (0)	3% (4)	97% (133)	2	9	Strong consensus
20. In acute and superficial dermatologic conditions, Phovia^®^ treatment should be employed once or twice weekly for a minimum of 2 weeks. Such conditions include fold dermatitis; superficial pyoderma; acute wounds.	0% (0)	3.7% (5)	96.3% (131)	4	9	Strong consensus
21. Depending on an individual’s response and the chronicity of the condition, additional Phovia^®^ treatments may be required.	0% (0)	1.5% (2)	98.5% (133)	3	9	Strong consensus
22. For surgical incisions and wounds healing by primary intention (wound the edges are closely re-approximated), it is recommended to perform one Phovia^®^ treatment in the immediate postoperative period or the first day after surgery, then continue Phovia^®^ treatments until the wound is healed.	0.7% (1)	3.8% (5)	95.5% (126)	7	9	Strong consensus
23. All surgical sites and wounds healing by second intention (a gap is left between the edges of the wound) including, but not limited to, skin grafting; releasing incisions; local, regional, and free skin flaps; excisional biopsy sites; it is recommended to apply Phovia^®^ treatment on the initial day of presentation immediately after the standard wound preparation process (wound exudate management, cleaning, debridement, culture, etc.), then once or twice weekly until the wound is healed.	0% (0)	2.4% (3)	97.6% (124)	13	9	Strong consensus

The variability in the total number of voters (equal or below 152) for each statement accounts for the use of the “no opinion” option by voters and for veterinarians who may not have submitted their answer in time before the voting process was closed

*FLE*, Fluorescent Light Energy

**Table 4:** Statements and voting results of the *Pet Owner Information* chapter

Statements	Validity 1-2-3 (*N*)	Validity 4-5-6 (*N*)	Validity 7-8-9 (*N*)	No opinion	Median	Results
Pet Owner Information						
27. Pet owners should be informed that Phovia^®^ is safe, usually painless, promotes wound healing, decreases inflammation, and lowers the potential for antibiotic resistance by reducing the time to clinical resolution.	0% (0)	1.5% (2)	98.5% (135)	1	9	Strong consensus
28. Although pets may experience mild discomfort, pet owners should be informed that pets treated with Phovia^®^ experience minimal side effects such as skin redness, itching, edema, small bruises, and burns; and the treatment reduces the amount of medication/topical treatments required at home.	0% (0)	3.6% (5)	96.4% (133)	1	9	Strong consensus
29. Pet owners should be informed that pets treated with Phovia^®^ may develop pink-colored coat and skin in the periphery of the treatment area due to the chromophore gel and that this does not represent a risk for the pet.	0.7% (1)	3.5% (5)	95.8% (136)	0	9	Strong consensus
30. Pet owners should also be informed that attending all Phovia^®^ follow-up sessions recommended by the veterinarian is key to a positive outcome and that based on the clinical results, the frequency and duration of Phovia^®^ prescription may vary.	0% (0)	0.7% (1)	99.3% (142)	0	9	Strong consensus
31. Vets should inform pet owners that currently 2- to 4-week initial treatment courses are required for most cutaneous conditions treated with Phovia^®^ and that some treatment lengths may need to be extended based on lesion severity, chronicity and patient response.	0.8% (1)	0.8% (1)	98.4% (121)	2	9	Strong consensus
32. Pet owners should be notified that there are currently no contraindications for use of Phovia^®^ with non-photosensitizing topical (e.g., flea/parasite products, shampoos) or systemic therapies.	2.9% (4)	2.1% (3)	95% (132)	2	9	Strong consensus
33. Pet owners should be encouraged to keep the Phovia^®^ treated areas clean and dry between treatments.	0.7% (1)	3.6% (5)	95.7% (133)	5	9	Strong consensus

The variability in the total number of voters (equal or below 152) for each statement accounts for the use of the “no opinion” option by voters and for veterinarians who may not have submitted their answer in time before the voting process was closed

## Photobiomodulation

All voters recognized that photobiomodulation via FLE could be used in veterinary skin conditions and agreed on its mode of action (Statements 1 to 3 with a *strong consensus*) (Table 2). The conditions of use of photobiomodulation via FLE (Statements 4 to 14) also were agreed upon. Voters acknowledged that after a dermatologic workup to identify any underlying etiologies, FLE should be implemented as early as possible in the disease process, either as a monotherapy or adjunct therapy to promote clinical outcomes. Voters concurred that photobiomodulation via FLE can reduce the reliance on antibiotic use (Statement 4) and acknowledged the recommendations for photobiomodulation to obtain the most favorable results (Statement 7).

**Table:** 

Box 1: *Statement 7*
For the most favorable results using photobiomodulation via fluorescent light energy: • Illuminate the treated area with a licensed LED lamp delivering a peak wavelength between 440-460 nm, with a power density of 55-129 mW/cm^2^ for 2 minutes at a maximum distance (~ 5 cm) [8, 17, 21, 31–39]. • Illuminate twice weekly or once weekly with 2 consecutive applications with cleaning and reapplication of new chromophore gel in between [8, 17, 21, 31–40]

### Indications and protocols

Veterinarians approved the indications in which photobiomodulation using FLE (Phovia^®^, Vetoquinol, France) could be used as a therapeutic modality with *strong consensus* (Statements 15 to 18) (Table 3). The variability of treatment duration using FLE (Phovia^®^, Vetoquinol, France) (Statements 19 to 21, 25, 26) ranged from once or twice weekly sessions for a minimum duration of two or four weeks depending on the dermatologic conditions treated (Table 3). Voters also consented to the protocols and duration of treatment of surgical incisions and wound healing by primary, second, and tertiary intention when using FLE (Phovia^®^, Vetoquinol, France) (statements 22 to 24) (Table 3).

**Table:** 

**Box 2**
*Statement 16*. Based on scientific studies* and case reports**, Phovia®as a monotherapy or adjunct treatment is recommended for the following indications^†^: • Interdigital pyoderma* [17, 31, 32, 36] • Surgical wounds* [41, 42] • Deep pyoderma* [17, 32, 43] • Superficial pyoderma* [20, 38] • Inflammatory perianal furunculosis (“fistula”)*, ** [20, 44] • Acute traumatic and chronic wounds*, ** [20, 45, 46] • Acute pyotraumatic dermatitis*,** [20, 33] ^*†*^*listed by level of evidence*
*Statement 17*. Based on the experience of a group of veterinary dermatologists, **Phovia® **can also be used as a monotherapy or adjunct therapy in the following indications: • Callus dermatitis • Scrotal dermatitis • Fold dermatitis (intertrigo) • Acral lick dermatitis

## Pet owner information

Attending veterinarians agreed that animal owners should be reminded that FLE (Phovia^®^, Vetoquinol, France) presents no contraindications when combined with non-photosensitizing topical or systemic treatments, that it is safe for both users and animals, and that side effects may include transient discomfort at the wound site or temporary hair colour change, which are considered risk-free for the animal (Statements 27 to 29, 32) (Table 4). Voters also acknowledged the importance of educating animal owners on the necessity of attending all scheduled treatment sessions for the animal to maximize FLE’s benefits (Statements 30 and 31) (Table 4).

## Discussion

The Delphi method has gained popularity over the past years as a method to identify and achieve areas of consensus among experts across a wide variety of health and non-health related fields [28, 29, 40]. Our study is the first consensus research using this method to establish recommendations for best practices for the use of FLE in veterinary dermatology and dermatologic surgery.

More than 150 veterinarians from nine countries took part in this study. This paper only presents and discusses the results from the first three chapters of the study, namely “Photobiomodulation”, “Indications and Protocols”, and “Pet Owner Information”. All 33 statements from these chapters of the study achieved a *great consensus* among participating veterinarians. The sections on “Practice Organization” and “Business Considerations” will be published separately because they delve deeper into the technical aspects of implementing FLE in one’s clinic and focus less on the scientific features behind FLE.

During the analysis of the results, we evaluated differences in the votes of the most experienced FLE (Phovia^®^, Vetoquinol, France) users within the voting group, i.e., more than five patients treated with the lamp, as compared to the votes of the group as a whole. When accounting only for these experienced users, 32 out of 33 statements reached a *strong consensus* with one statement reaching a *good consensus* (≥ 75% of votes ≥ 7 or median ≥ 8) (*data not shown*). While these results do not institute an absolute truth on the subject, they showcase a noteworthy degree of consensus that can be reached among veterinary experts. Consensus within the selected expert panel was reached despite the geographical variability of voters, diverse FLE (Phovia^®^, Vetoquinol, France) use frequencies, and differences between types of practices.

The consensus appears consistent with the thorough scrutiny conducted by the international SC, which meticulously screened and reviewed each statement of the study before incorporating it into the final set of recommendations. It also reflects an existing understanding among experts regarding the mode of action, conditions of use, and application of FLE in veterinary dermatology. Despite the global consensus achieved, the recommendations established by the SC are practical and need to be adapted based on the local specificities of each country and practice.

Over 95% of voters (median score of 9) agreed that veterinarians should be encouraged to use photobiomodulation via FLE to improve clinical outcomes in their patients and reduce reliance on systemic or even topical antibiotics, ultimately promoting antibiotic stewardship. Indeed, one notable application of FLE is its use in the management of bacterial skin infections [8]. Photobiomodulation exerts its antimicrobial effect by damaging bacterial cell walls, disrupting biofilm, and suppressing resistant gene expression while simultaneously enhancing the immune response, all of which help to reduce antibiotic use in these cases [17, 36, 38, 43]. This is supported by findings of a recent meta-analysis that concluded there is good scientific evidence identified for the recommendation of fluorescence biomodulation (FBM) aka FLE for the management of canine interdigital pyoderma and canine deep pyoderma in combination with systemic antibiotic therapy [8]. FLE also has demonstrated promise in promoting tissue repair, reducing inflammation, accelerating wound healing, and reducing treatment times by at least 50% [8, 38, 43].

The application of FLE is painless, and in most animals, sedation is not required. However, in anxious or aggressive patients, mild sedation may be considered to facilitate application. From a safety perspective, FLE has been extensively studied, and no significant adverse effects have been reported in clinical studies or expert clinical experience. Nevertheless, veterinarians should inform pet owners that mild, transient side effects, such as localized skin redness, itching, edema, burn or small bruises, may occasionally occur.

The use of FLE as a monotherapy versus an adjunct therapy to improve animal welfare is a subject of considerable interest and ongoing investigation [20, 38, 43]. As a monotherapy, FLE has been shown to be beneficial in certain dermatological conditions, including interdigital furunculosis, pyotraumatic dermatitis, superficial and deep pyoderma, and traumatic wounds [8, 17, 20, 31–33, 36, 38, 43]. Additionally, as an adjunctive treatment to systemic antibiotics, it has demonstrated efficacy in cases of deep pyoderma and interdigital furunculosis, where combination therapy may enhance clinical outcomes and potentially reducing the duration of antimicrobial use.

The decision to use FLE as a monotherapy or as part of an adjunctive treatment approach should be guided by the severity and chronicity of the condition, the clinical judgment of the veterinarian, and pet owner compliance and adherence to treatment protocols. As an adjunct to other therapeutic modalities, such as topical medications or systemic therapies, FLE can synergistically enhance the overall therapeutic effect, potentially reducing the time to clinical resolution and improving both animal welfare and owner quality of life [8, 20].

FLE can also improve the quality of wound healing as an adjunct to conventional post-operative care. Salvaggio et al., 2020’s study demonstrated the positive effect of FLE at the microscopic level with areas of canine surgical wounds treated with FLE achieved lower histology scores consistent with re-epithelization, decreased dermal inflammation and improved matrix formation [41]. Marchegiani et al., 2024’s prospective, blinded clinical trial on the management of surgical incisions after mastectomy in female dogs demonstrated the positive impact of FLE at the macroscopic level [42]. More similar prospective, placebo-controlled studies would strengthen the existing evidence of FLE’s capacity to enhance clinical outcomes in veterinary dermatology.

Experienced FLE users who have witnessed its clinical efficacy firsthand might be more inclined to implement FLE in a variety of dermatological conditions. Nevertheless, an accurate and thorough diagnostic assessment to identify the underlying etiology, a clear understanding of the indications where photobiomodulation via FLE is successful, and knowledge of the appropriate treatment duration for each indication (acute vs. chronic) are essential before recommending FLE therapy [17]. Indeed, less experienced veterinarians who fail to see concrete results when using FLE due to poor case implementation or improper technique may assume that the treatment modality does not work and will not persevere with its use and revert to traditional therapeutic options that they are more familiar and confident with.

FLE is a versatile therapy applicable to various skin conditions, wounds, and inflammatory skin diseases. When first incorporating FLE into clinical practice, it is practical to begin with conditions that typically respond rapidly and predictably to treatment, such as superficial wounds, superficial pyoderma, or acute pyotraumatic dermatitis. These cases allow the clinician to become familiar with treatment technique, patient positioning, and expected healing patterns. Once confidence with the method is established, FLE can be readily applied to more complex, chronic, or multifactorial conditions (e.g., perianal fistulas, deep pyoderma, or extensive chronic wounds), which may require longer treatment courses and multimodal management [43–45]. For chronic wounds and dermatological problems that warrant further investigation (e.g., calcinosis cutis, interdigital furunculosis), FLE treatment protocols require more time to demonstrate efficacy and necessitate simultaneous treatment of the primary condition with a multimodal approach (e.g. topical and/or systemic therapies) [31, 35]. As practitioners implement FLE and witness the benefits of its applications, their confidence and belief in its efficacy are likely to increase. FLE treatment should generally be stopped once the lesion has fully healed, as observed in acute and superficial dermatologic conditions such as, superficial pyoderma, acute wounds, or surgical wounds. For chronic and deep dermatological conditions, such as deep pyoderma, interdigital furunculosis, or inflammatory perianal furunculosis, which typically require at least four weeks of treatment, lack of improvement after this period should prompt discontinuation and re-evaluation of the underlying condition. Additionally, treatment should be stopped if significant adverse effects occur, such as unexpected skin reactions or discomfort, or if poor compliance with the prescribed protocol (e.g., missed sessions or irregular treatment application) is observed, as this may compromise efficacy.

Before initiating FLE therapy in an animal, the veterinarian should strongly consider explaining the benefits of photobiomodulation to the animal owner, including accelerated wound healing, reduced inflammation, and its potential to minimize antibiotic use. By engaging in open and transparent communication with animal owners, veterinarians will foster trust and empower owners to make informed decisions about their animal’s well-being. This, in turn, will ultimately increase the owner’s adherence with the treatment plans [7]. Discussing the noninvasiveness, ease of application, safety profile of FLE, and lack of contraindications when combined with non-photosensitizing topical and systemic therapies will reassure owners about the well-being of their animals during treatment. Dissemination of key information on the use of FLE in veterinary dermatology will educate animal owners about this recent cutting-edge therapeutic modality for their animals and will strengthen the veterinarian-owner bond, which facilitates animal welfare.

Although the Delphi method is known and followed as a structured procedure, this approach presents some limitations associated with voter profiles, statement development, and criteria to achieve consensus [24–26]. Our study sought to limit these potential biases as much as possible to achieve maximum objectivity. Nevertheless, the choice of knowledgeable voters, a nonrandom sampling process due to the need to ensure a certain level of expertise in the study, is an inherent bias in the process itself [24–26, 47]. Voters from all nine participating countries were selected on objective criteria based on their use of FLE (Phovia^®^ system, Vetoquinol) and their experience with using the system. These criteria made it possible to obtain a sample with encouraging characteristics: a median of five pets treated with FLE per month for 54% of voting veterinarians. The absence of any remuneration testified to their commitment.

Regarding the statements, an initial cross-examination of the literature by the SC allowed for the identification of the main questions encountered in veterinary clinical practice and facilitated precise wording. As for the threshold used to establish consensus, our study adhered to a rigorous approach incorporating two specific criteria: the overall percentage of scores ≥ 7 and the median score ≥ 8. This stringent and exacting definition enhances the credibility of our results. Furthermore, our research maintained a consistent and thorough separation between veterinarians participating in completely anonymous voting and members of the SC abstaining from the voting process. In line with typical Delphi consensus, this project ultimately seeks to provide veterinarians with a practical guide while empowering them to retain control over their practice and tailor it to individual pet circumstances.

## Conclusion

Throughout this intercontinental consensus study utilizing the Delphi method, all 33 statements outlined in this paper achieved a *strong consensus* from a cohort of 152 veterinarians representing nine countries. This result not only showcases the existing knowledge among veterinarians regarding the mechanism of action of FLE but also establishes a foundation for the integration of photobiomodulation via FLE in veterinary dermatology, which represents a field where its benefits have been extensively demonstrated. When applied early in the disease process, photobiomodulation via FLE enhances the clinical outcomes of an animal’s treatment regimen notably reducing healing times by a minimum of 50%. This non-invasive, user-friendly, and safe therapy poses no contraindications when combined with non-photosensitizing therapies. The versatility of photobiomodulation via FLE is evident, proving successful across a variety of veterinary dermatological pathologies provided the length of treatment aligns with the chronicity and severity of the condition. In the absence of established recommendations for FLE use in treating animals, this consensus serves as a pivotal step in formalizing best practice guidelines for veterinarians. Ultimately, this consensus research will drive the utilization of FLE in addressing dermatological conditions, thereby optimizing the welfare of both animals and their owners.

## Supplementary Information

Supplementary Material 1.

## Data Availability

Data and methodology presented in the study are included in the article/supplementary material, further inquiries can be directed to the corresponding author(s).
