# Supplementary material for: Clinical practice recommendations and expected outcomes with fluorescent light energy: a Delphi-like consensus
Source: BMC Vet Res. 2025 Oct 2;21:565. doi: 10.1186/s12917-025-05047-6 (PMC12492513; doi:10.1186/s12917-025-05047-6)
Supplement: Supplementary file 1 — Supplementary Material 1. [file 12917_2025_5047_MOESM1_ESM.docx]

**Questionnaire: Delphi consensus questionnaire on the clinical use of Fluorescent Light Energy (FLE) in veterinary dermatology**

Please rate each statement on a scale from 1 to 9, where 1 = strongly disagree and 9 = strongly agree. If you have no opinion on a statement, select “no opinion.”

**Photobiomodulation chapter**

1. ***Photobiomodulation via fluorescent light energy in veterinary skin disorders is a recommended supportive therapeutic approach for its regenerative, anti-inflammatory, and likely antimicrobial effects.***

How much do you agree with this statement?

1 2 3 4 5 6 7 8 9  No opinion
(1 = Strongly disagree  9 = Strongly agree)

1. ***Use of a chromophore gel with blue LED helps to promote the production of fluorescent light energy with a broader spectrum of visible light (440 to 700 nm) that penetrates to greater depths in the skin (up to approximately 6 mm), providing a broader range of effects.***

How much do you agree with this statement?

1 2 3 4 5 6 7 8 9  No opinion
(1 = Strongly disagree  9 = Strongly agree)

1. ***In the skin, light absorption generated by fluorescent light energy depends on the interaction with the different chromophores (e.g. cytochrome C oxidase, flavins, opsins) which absorb specific wavelengths to exert their biologic effects.***

How much do you agree with this statement?

1 2 3 4 5 6 7 8 9  No opinion
(1 = Strongly disagree  9 = Strongly agree)

1. ***Veterinary practitioners should be encouraged to use photobiomodulation via fluorescent light energy to promote clinical outcomes and reduce the length of systemic antibiotic use in patients with bacterial pyoderma, including patients with MRSP.***

How much do you agree with this statement?

1 2 3 4 5 6 7 8 9  No opinion
(1 = Strongly disagree  9 = Strongly agree)

1. ***Photobiomodulation via fluorescent light energy can be used as a monotherapy or as an adjunct to support other therapeutic regimens to manage non-neoplastic dermatologic conditions.***

How much do you agree with this statement?

1 2 3 4 5 6 7 8 9  No opinion
(1 = Strongly disagree  9 = Strongly agree)

1. ***Photobiomodulation via fluorescent light energy is recommended to be used early in the disease process to maximize efficacy.***

How much do you agree with this statement?

1 2 3 4 5 6 7 8 9  No opinion
(1 = Strongly disagree  9 = Strongly agree)

1. ***For the most favorable results using photobiomodulation via fluorescent light energy:***

- ***illuminate the treated area with a licensed LED lamp delivering a peak wavelength 440–460 nm, power density 55–129 mW/cm² for 2 minutes at a maximum distance ~5 cm;***
- ***illuminate twice weekly or once weekly with 2 consecutive applications, with cleaning and reapplication of new chromophore gel between applications.***

How much do you agree with this statement?

1 2 3 4 5 6 7 8 9  No opinion
(1 = Strongly disagree  9 = Strongly agree)

1. ***To maximize efficacy and increase the likelihood of photonic penetration during photobiomodulation via fluorescent light energy, it is recommended to clip hair from treatment areas.***

How much do you agree with this statement?

1 2 3 4 5 6 7 8 9  No opinion
(1 = Strongly disagree  9 = Strongly agree)

1. ***To achieve optimal effects, photobiomodulation via fluorescent light energy should be used with a photoconverter gel uniformly applied to an approximately 2 mm layer of the treatment areas.***

How much do you agree with this statement?

1 2 3 4 5 6 7 8 9  No opinion
(1 = Strongly disagree  9 = Strongly agree)

1. ***Once fluorescent light energy treatment is completed, the photoconverter gel must be cleansed from the treated site(s).***

How much do you agree with this statement?

1 2 3 4 5 6 7 8 9  No opinion
(1 = Strongly disagree  9 = Strongly agree)

1. ***When initiating fluorescent light energy treatment, a dermatologic workup is recommended to identify and treat all underlying causes and associated factors.***

How much do you agree with this statement?

1 2 3 4 5 6 7 8 9  No opinion
(1 = Strongly disagree  9 = Strongly agree)

1. ***An incomplete response to fluorescent light energy after 2 weeks of treatment should prompt reevaluation of the underlying etiology and the therapeutic regimen.***

How much do you agree with this statement?

1 2 3 4 5 6 7 8 9  No opinion
(1 = Strongly disagree  9 = Strongly agree)

1. ***Photobiomodulation via fluorescent light energy treatment for a surface area exceeding the lamp diameter will require multiple treatments, increasing the length of time per session, or the use of multiple lamps being used simultaneously to keep session lengths to a minimum.***

How much do you agree with this statement?

1 2 3 4 5 6 7 8 9  No opinion
(1 = Strongly disagree  9 = Strongly agree)

1. ***Fluorescent light energy is painless; sedation may be needed for aggressive or anxious patients.***

How much do you agree with this statement?

1 2 3 4 5 6 7 8 9  No opinion
(1 = Strongly disagree  9 = Strongly agree)

**Indications and Protocols chapter**

1. ***Photobiomodulation via fluorescent light energy is recommended for skin infections, wounds, and chronic/recurrent inflammatory skin conditions as it promotes tissue healing, reduces inflammation, and supports collagen production.***

How much do you agree with this statement?

1 2 3 4 5 6 7 8 9  No opinion
(1 = Strongly disagree  9 = Strongly agree)

1. ***Based on scientific studies* and case reports**, Phovia® as a monotherapy or adjunct treatment is recommended for the following indications†:***

- ***Interdigital pyoderma****
- ***Surgical wounds****
- ***Deep pyoderma****
- ***Superficial pyoderma****
- ***Inflammatory perianal furunculosis (“fistula”)****
- ***Acute traumatic and chronic wounds****
- ***Acute pyotraumatic dermatitis****

*^†^Listed by level of evidence*

How much do you agree with this statement?

1 2 3 4 5 6 7 8 9  No opinion
(1 = Strongly disagree  9 = Strongly agree)

1. ***Based on the experience of a group of veterinary dermatologists, Phovia® can also be used as a monotherapy or adjunct therapy in the following indications:***

- ***Callus dermatitis***
- ***Scrotal dermatitis***
- ***Fold dermatitis (intertrigo)***
- ***Acral lick dermatitis***

How much do you agree with this statement?

1 2 3 4 5 6 7 8 9  No opinion
(1 = Strongly disagree  9 = Strongly agree)

1. ***Based on single or few cases, Phovia® treatment may be indicated in the following conditions but requires further investigation:***

- ***Equine pastern dermatitis (pyoderma, leucocytoclastic vasculitis)***
- ***Actinic dermatitis***
- ***Feline idiopathic ulcerative dermatitis***
- ***Cutaneous vasculitis (ear tip vasculitis***
- ***Marsupialized abscesses***
- ***Follicular dysplasia (alopecia x, pattern alopecia, post-clipping alopecia, seasonal flank)***
- ***Calcinosis cutis***
- ***Pre- and post-surgical wounds (skin grafts, skin flaps, traumatic injuries).***

How much do you agree with this statement?

1 2 3 4 5 6 7 8 9  No opinion
(1 = Strongly disagree  9 = Strongly agree)

1. ***It is recommended that Phovia® 2-minute sessions be applied twice weekly or once weekly with 2 consecutive applications.***

How much do you agree with this statement?

1 2 3 4 5 6 7 8 9  No opinion
(1 = Strongly disagree  9 = Strongly agree)

1. ***In acute and superficial dermatologic conditions, Phovia® treatment should be employed once or twice weekly for a minimum of 2 weeks. Such conditions include:***

- ***Fold dermatitis***
- ***Superficial pyoderma***
- ***Acute wounds***

How much do you agree with this statement?

1 2 3 4 5 6 7 8 9  No opinion
(1 = Strongly disagree  9 = Strongly agree)

1. ***Depending on an individual’s response and the chronicity of the condition, additional Phovia® treatments may be required.***

How much do you agree with this statement?

1 2 3 4 5 6 7 8 9  No opinion
(1 = Strongly disagree  9 = Strongly agree)

1. ***For surgical incisions and wounds healing by primary intention (wound the edges are closely re-approximated), it is recommended to perform one Phovia® treatment in the immediate postoperative period or the first day after surgery, then continue Phovia® treatments until the wound is healed.***

How much do you agree with this statement?

1 2 3 4 5 6 7 8 9  No opinion
(1 = Strongly disagree  9 = Strongly agree)

1. ***All surgical sites and wounds healing by second intention (a gap is left between the edges of the wound) including, but not limited to, skin grafting; releasing incisions; local, regional, and free skin flaps; excisional biopsy sites; it is recommended to apply Phovia® treatment on the initial day of presentation immediately after the standard wound preparation process (wound exudate management, cleaning, debridement, culture, etc.), then once or twice weekly until the wound is healed.***

How much do you agree with this statement?

1 2 3 4 5 6 7 8 9  No opinion
(1 = Strongly disagree  9 = Strongly agree)

1. ***All surgical sites and wounds healing by tertiary healing (third intention - primary closure is delayed by 4–6 days), it is recommended to apply Phovia® treatment on the initial day of primary closure then once or twice weekly until the wound is healed***

How much do you agree with this statement?

1 2 3 4 5 6 7 8 9  No opinion
(1 = Strongly disagree  9 = Strongly agree)

1. ***For deep and chronic dermatitis, it is recommended to use Phovia® once or twice weekly for a minimum of 4 weeks. Such conditions include:***

- ***Callus dermatitis***
- ***Pedal furunculosis***
- ***Inflammatory perianal furunculosis (“fistula”)***

How much do you agree with this statement?

1 2 3 4 5 6 7 8 9  No opinion
(1 = Strongly disagree  9 = Strongly agree)

1. ***After a successful outcome with Phovia®, repeated treatments can be used to address any relapsing lesions using either the same protocol if lesions have returned to their original presentation OR using a modified pulse protocol monthly to twice monthly pending the severity of the lesions and response to fluorescent light energy exposure.***

How much do you agree with this statement?

1 2 3 4 5 6 7 8 9  No opinion
(1 = Strongly disagree  9 = Strongly agree)

***Pet Owner Information* chapter**

1. ***Pet owners should be informed that Phovia® is safe, usually painless, promotes wound healing, decreases inflammation, and lowers the potential for antibiotic resistance by reducing the time to clinical resolution.***

How much do you agree with this statement?

1 2 3 4 5 6 7 8 9  No opinion
(1 = Strongly disagree  9 = Strongly agree)

1. ***Although pets may experience mild discomfort, pet owners should be informed that pets treated with Phovia® experience minimal side effects such as skin redness, itching, edema, small bruises, and burns; and the treatment reduces the amount of medication/topical treatments required at home.***

How much do you agree with this statement?

1 2 3 4 5 6 7 8 9  No opinion
(1 = Strongly disagree  9 = Strongly agree)

1. ***Pet owners should be informed that pets treated with Phovia® may develop pink-colored coat and skin in the periphery of the treatment area due to the chromophore gel and that this does not represent a risk for the pet.***

How much do you agree with this statement?

1 2 3 4 5 6 7 8 9  No opinion
(1 = Strongly disagree  9 = Strongly agree)

1. ***Pet owners should also be informed that attending all Phovia® follow-up sessions recommended by the veterinarian is key to a positive outcome
   and that based on the clinical results, the frequency and duration of Phovia® prescription may vary.***

How much do you agree with this statement?

1 2 3 4 5 6 7 8 9  No opinion
(1 = Strongly disagree  9 = Strongly agree)

1. ***Vets should inform pet owners that currently 2- to 4-week initial treatment courses are required for most cutaneous conditions treated with Phovia®
   and that some treatment lengths may need to be extended based on lesion severity, chronicity and patient response.***

How much do you agree with this statement?

1 2 3 4 5 6 7 8 9  No opinion
(1 = Strongly disagree  9 = Strongly agree)

1. ***Pet owners should be notified that there are currently no contraindications for use of Phovia® with non-photosensitizing topical (e.g., flea/parasite products, shampoos) or systemic therapies.***

How much do you agree with this statement?

1 2 3 4 5 6 7 8 9  No opinion
(1 = Strongly disagree  9 = Strongly agree)

1. ***Pet owners should be encouraged to keep the Phovia® treated areas clean and dry between treatments.***

How much do you agree with this statement?

1 2 3 4 5 6 7 8 9  No opinion
(1 = Strongly disagree  9 = Strongly agree)
